# Supplementary material for: Efficacy of a school-based mental health intervention among Zambian youth: a cluster-randomized controlled trial
Source: Glob Ment Health (Camb). 2025 Mar 31;12:e43. doi: 10.1017/gmh.2025.33 (PMC12037358; doi:10.1017/gmh.2025.33)
Supplement: Saasa et al. supplementary material [file S2054425125000330sup001.zip › Figure 8.docx]

**Figure 8.** Means of positive parent-child relationships over time. T0 = Baseline, T3 = 3-Month Follow-Up.
