# Supplementary material for: Efficacy of a school-based mental health intervention among Zambian youth: a cluster-randomized controlled trial
Source: Glob Ment Health (Camb). 2025 Mar 31;12:e43. doi: 10.1017/gmh.2025.33 (PMC12037358; doi:10.1017/gmh.2025.33)
Supplement: Saasa et al. supplementary material [file S2054425125000330sup001.zip › Figure 4.docx]

**Figure 4.** Means of depression over time. T0 = Baseline; T1 = Post-Intervention for tx group, Second Baseline for waitlist control group; T2 = Post- Intervention for waitlist control group; T3 = 3-Month Follow-Up.
